# Clinical perception and novel insights of chronic myelomonocytic leukemia: a 10-year multi-center retrospective study

**DOI:** 10.3389/fmed.2025.1711684

**Published:** 2026-01-15

**Authors:** Yanquan Liu, Zhimin Yan, Jingdong Zhang, Xiaojun Chen, Jianzhen Shen, Honghua He, Yue Yin, Huidong Guo, Fanlin Zeng

**Affiliations:** 1 Jiangxi Health Commission Key Laboratory of Leukemia, Department of Hematology, The Affiliated Ganzhou Hospital (Ganzhou People’s Hospital), Jiangxi Medical College, Nanchang University, Ganzhou, Jiangxi, China; 2 Department of Hematology, The First Affiliated Hospital of Gannan Medical University, Ganzhou, Jiangxi, China; 3 Department of Hematology, The Affiliated Hospital of Putian University, Putian, Fujian, China; 4 Fujian Institute of Hematology, National Key Laboratory of Hematology, Fujian Medical University Union Hospital, Fuzhou, Fujian, China; 5 Department of Hematology, The Affiliated Hospital (First School of Clinical Medicine) of Guangdong Medical University, Zhanjiang, Guangdong, China; 6 The First Affiliated Hospital (First Clinical Medical College) of Gannan Medical University, Ganzhou, Jiangxi, China

**Keywords:** chronic myelomonocytic leukemia, clinical features, diagnosis and differential diagnosis, myeloid neoplasm, prognosis, treatment advances

## Abstract

**Background and objective:**

Chronic myelomonocytic leukemia (CMML) is a malignant clonal disorder characterized by both myelodysplastic syndrome (MDS) and myeloproliferative neoplasm (MPN) features. Due to its relatively low incidence, there remains a lack of consensus regarding diagnostic criteria and therapeutic strategies within the academic community, which poses significant challenges in clinical management. This study aims to investigate the clinical manifestations, diagnostic approaches, therapeutic interventions, and prognostic factors associated with CMML, with the goal of providing evidence-based insights for future basic research and clinical practice in the field of hematology.

**Methods:**

Clinical data from 271 CMML patients treated at five tertiary hospitals between January 2015 and May 2025 were collected and analyzed. The clinical characteristics, treatment modalities, and outcomes were systematically reviewed. Comprehensive prognostic evaluation was conducted using the Kaplan–Meier method, Log-rank test, and Cox proportional hazards regression model.

**Results:**

A total of 271 CMML patients were enrolled, including 178 males (65.68%) and 93 females (34.32%), with a median age at diagnosis of 66 years (range: 26–89). According to the FAB classification, 109 cases (40.22%) were classified as myelodysplastic-type CMML (MD-CMML), and 162 cases (59.78%) as myeloproliferative-type CMML (MP-CMML). Based on the WHO classification, the distribution was as follows: 59 cases (21.77%) of CMML-0, 66 cases (24.35%) of CMML-1, and 146 cases (53.87%) of CMML-2. First-line treatment primarily involved chemotherapy, while 107 patients received only supportive care. Treatment response was evaluable in 199 patients: 97 cases achieved complete remission (CR), 63 cases achieved partial remission (PR), 32 cases had stable disease (SD), and 7 cases experienced disease progression (PD). Follow-up was completed by June 30, 2025. Among the 271 patients, 159 cases (58.67%) were alive, 97 cases (35.79%) had died, and 15 cases (5.54%) were lost to follow-up. The median overall survival (OS) was 23.5 months (range: 0.5–109). Multivariate analysis identified that factors associated with poor OS included elevated neutrophil count, increased monocyte count, decreased hemoglobin (HB) levels, elevated lactate dehydrogenase (LDH), increased β2-microglobulin (β2-MG), and peripheral blood blast count ≥5% (*p* < 0.05), while the decreased HB and peripheral blood blast count ≥5% was independent adverse prognostic factors for OS.

**Conclusion:**

CMML is a highly heterogeneous disease with generally unfavorable clinical outcomes. Although chemotherapy can induce remission in some cases, long-term survival remains limited. The enrollment in clinical trials should be encouraged to improve patient prognosis.

## 1 Introduction

As a relatively rare non-solid malignant clonal disorder originating from the bone marrow lymphohematopoietic system, chronic myelomonocytic leukemia (CMML) is typically characterized by persistent monocytosis in the peripheral blood. It may also present with dysplastic hematopoiesis or bone marrow fibrosis (1, 2). According to the 2008 World Health Organization (WHO) classification of tumors of hematopoietic and lymphoid tissues, CMML is categorized under myelodysplastic/myeloproliferative neoplasms (MDS/MPN), moreover, CMML possesses the clinical, molecular biological and cytogenetic characteristics of both MDS and MPN, reflecting its hybrid features of both MDS and MPN (3–5).

To date, consensus on standardized diagnostic and therapeutic guidelines for CMML remains lacking in the fields of hematology and oncology. In particular, domestic and international research on CMML has predominantly consisted of single-center case reports, with limited multi-center, large-sample retrospective studies. As a result, the understanding and clinical management of CMML remain insufficient. To address this gap, this study retrospectively analyzed clinical data from 271 CMML patients treated at five tertiary hospitals over the past decade. The objective was to explore the clinical characteristics, diagnostic and therapeutic strategies, and prognostic factors of CMML, with the aim of providing reference for future basic and clinical research in this field.

## 2 Materials and methods

### 2.1 Study population and clinical data

**Figure 1:**
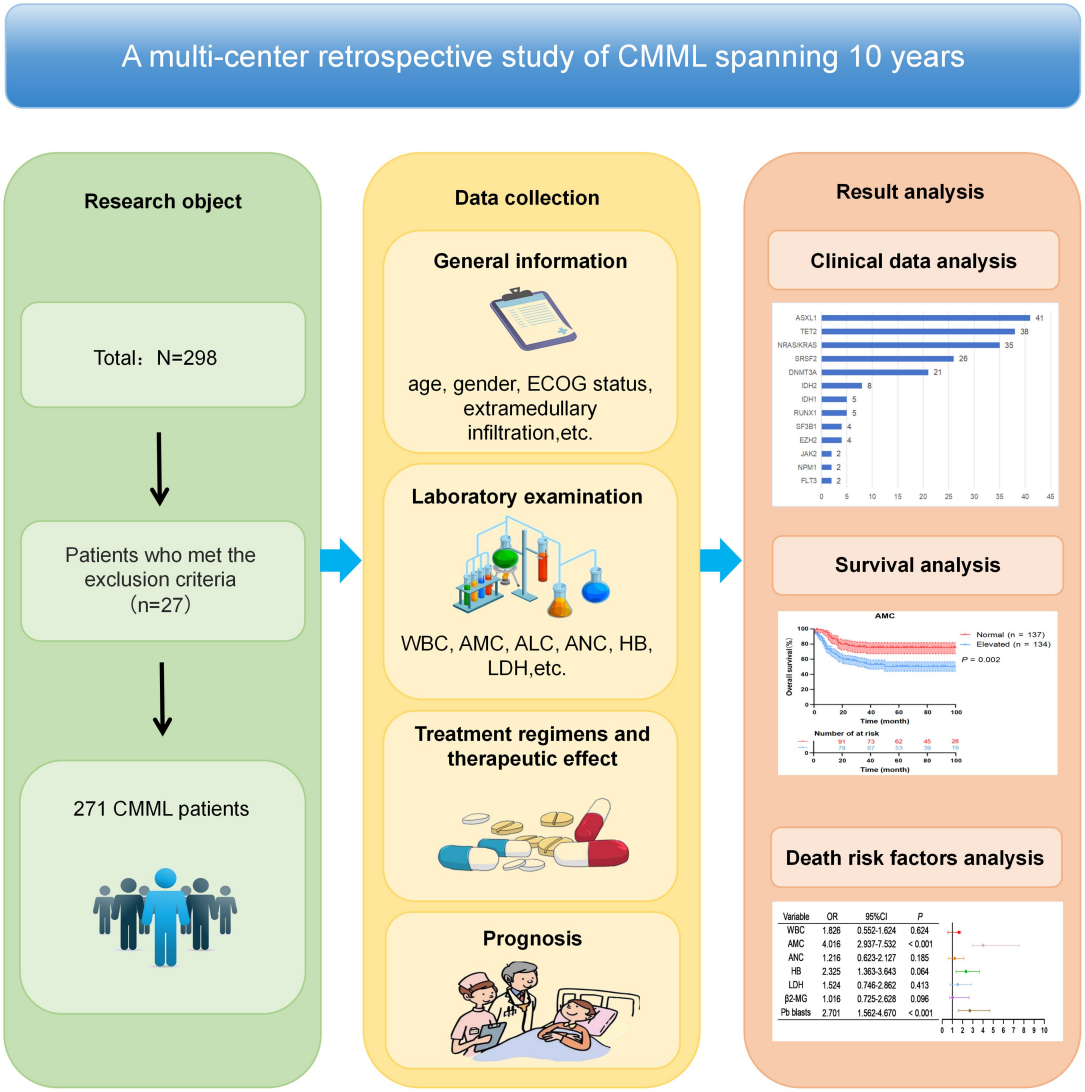
The detailed methodology of this research.

A total of 271 CMML patients diagnosed according to MICM (morphology, immunology, cytogenetics, and molecular biology) criteria were enrolled from the hematology departments of five tertiary hospitals: the Affiliated Ganzhou Hospital of Nanchang University, Fujian Medical University Union Hospital, Affiliated Hospital of Putian University, Affiliated Hospital of Guangdong Medical University, and the First Affiliated Hospital of Gannan Medical University, between January 2015 and May 2025. All diagnoses were consistent with the WHO diagnostic criteria for CMML from the 2008 and 2016 editions (3, 4, 6). According to the WHO classification, CMML is categorized as follows: (1) CMML-0: Peripheral blood blast count (PBC) < 2% and/or bone marrow PBC < 5%; (2) CMML-1: Peripheral blood PBC 2–4% and/or bone marrow PBC 5–9%; (3) CMML-2: Peripheral blood PBC 5–19%, bone marrow PBC 10–19%, and/or presence of Auer rods. Additionally, CMML can be classified according to the FAB criteria (7): (1) Myelodysplastic-type CMML: WBC < 13 × 10^9^/L; (2) Myeloproliferative-type CMML: WBC ≥ 13 × 10^9^/L. This study applied the M.D. Anderson Prognostic Scoring System (MDAPSS) and the CMML-specific Prognostic Scoring System (CPSS) to stratify the risk of enrolled patients. The detailed methodology is illustrated in Figure 1.

### 2.2 Data analysis and measured parameters

Clinical data and laboratory indicators were systematically collected and analyzed, including age, gender, occupational history, lifestyle background, comorbidities, initial symptoms or signs, presence of hepatosplenomegaly or lymphadenopathy, red blood cell (RBC) transfusion dependence, peripheral blood cell counts (multilineage involvement), lactate dehydrogenase (LDH), peripheral blood to bone marrow blast ratio, β2-microglobulin (β2-MG), serum albumin, neutrophil alkaline phosphatase (NAP) score, bone marrow dysplasia, degree of myelofibrosis, cytogenetic (karyotype) findings, and molecular (gene mutation) test results. Dysplasia of bone marrow (DBM) was diagnosed according to the 2008 WHO consensus criteria (3, 4), and myelofibrosis was assessed based on the 2016 WHO classification (6). RBC transfusion dependence (5) was defined as the requirement for at least two units of red blood cell transfusions within the past month due to anemia with Hb < 85 g/L to alleviate clinical symptoms. It should be noted that the diagnosis of DBM requires the presence of ≥10% abnormal hematopoietic cells in the bone marrow smear. Bone marrow biopsy findings may additionally inform the assessment of marrow architecture or fibrosis. In cases where discrepancies exist between smear and biopsy results, it is recommended to perform a comprehensive evaluation incorporating genetic testing and clinical context. If warranted, repeat sampling should be considered.

### 2.3 Morphologic and histological analysis

All CMML patients included in this study underwent bone marrow puncture biopsy, bone marrow smear was examined through morphologic assessment (Wright-Giemsa staining or other cytochemical staining methods). And the bone marrow biopsy tissue was stained by immunohistochemistry (IHC). Bone marrow slides were reviewed for multilineage dysplasia and abnormal maturation, and if available, reticulin and collagen stains were reviewed or performed to grade bone marrow fibrosis (MF-0 to MF-3) per standardized guidelines. Furthermore, approximately 0.5–1 cm of bone marrow biopsy tissue was embedded in paraffin, and sectioned. These sections were subsequently stained with hematoxylin and eosin (H&E), dehydrated, and mounted for examination. Histopathological changes were evaluated and scored using an optical microscope.

### 2.4 Therapeutic medications and regimens

The CMML patients included in this study were comprehensively assessed based on baseline data, including general health status, age, history of underlying diseases and comorbidities, disease risk stratification, physical tolerance, treatment preference, and economic considerations. Treatment regimens were formulated in accordance with established domestic and international guidelines. The primary therapeutic approaches for CMML patients in this study included symptomatic and supportive care (e.g., hydroxyurea for leukocytosis, alkalization, hydration), chemotherapy, and epigenetic agents [such as hypomethylating agents (HMAs), including azacitidine (AZA), decitabine (DAC), among others], administered as monotherapy or in combination with chemotherapy. For certain patients, treatment plans, drug selection, dosages, and administration routes were adjusted in real time and tailored to individual characteristics, including age, physical and psychological condition, and comorbidities. Only 13 patients in this study underwent allogeneic hematopoietic stem cell transplantation (allo-HSCT).

### 2.5 Therapeutic efficacy criteria

This study evaluated the therapeutic response of CMML patients who completed at least four full treatment cycles and had complete clinical documentation. The efficacy outcomes included complete remission (CR), partial remission (PR), complete cytogenetic response (CCyR), optimal marrow response (OMR), partial marrow response (PMR), clinical benefit (CB), and progressive disease (PD). Additionally, cases not meeting the aforementioned response criteria were classified as stable disease (SD), in accordance with the MDS/MPN response criteria. The overall response rate (ORR) was calculated as the sum of CR, PR, OMR, PMR, and CB (5).

### 2.6 Prognostic follow-up

All participating medical centers conducted individualized follow-up assessments for CMML patients through electronic medical records, supplemented by telephone interviews and other follow-up methods. The follow-up period concluded on June 30, 2025. Prognostic evaluation primarily focused on overall survival (OS) and leukemia-free survival (LFS). OS was defined as the time interval from CMML diagnosis to death from any cause or the end of follow-up. LFS was defined as the duration from diagnosis to leukemia transformation, death, or the end of follow-up.

### 2.7 Statistical analysis

Clinical data were analyzed using SPSS 26.0 statistical software. Normally distributed continuous variables were expressed as mean ± standard deviation (Mean ± SD), and compared using the *t*-test. Non-normally distributed data were presented as median (interquartile range) and analyzed using the Mann–Whitney *U* test. Categorical variables were expressed as frequency and percentage. Univariate survival analysis was performed using the Kaplan–Meier method and log-rank test. Variables showing statistical significance were further analyzed using the Cox proportional hazards regression model for multivariate analysis. Binary logistic regression was employed to identify risk factors associated with mortality. Survival curves and forest plots were generated using GraphPad Prism 9.0 software. The *p*-value < 0.05 was considered statistically significant.

## 3 Results

### 3.1 Clinical characteristics and manifestations

As shown in Table 1, based on inclusion and exclusion criteria and actual follow-up data, a total of 271 CMML patients were enrolled, including 178 males (65.68%) and 93 females (34.32%), yielding a male-to-female ratio of 1.91:1. The median age at diagnosis was 66 years (range: 26–89). The clinical manifestations of CMML were nonspecific and included fatigue, abnormal blood counts, fever, infection, abdominal discomfort, bleeding, and cutaneous abnormalities. Splenomegaly was observed in 159 patients, hepatomegaly in 21 cases, and lymphadenopathy in 66 cases. The median percentage of blasts in peripheral blood (PBC) was 2% (range: 0–14.5%), and in bone marrow was 7% (range: 3.5–19.2%). According to the FAB classification, 109 cases (40.22%) were classified as MD-CMML and 162 (59.78%) as MP-CMML. Based on the WHO classification, 59 cases (21.77%) were CMML-0, 66 cases (24.35%) were CMML-1, and 146 cases (53.87%) were CMML-2.

**Table 1:** Clinical characteristics and manifestations of 271 CMML patients.

Items and variables	Baseline distribution (*n* = 271)
Age (years) *n* (%)	
<60	166 (61.25)
≥60	105 (38.75)
Gender, *n* (%)	
Male	178 (65.68)
Female	93 (34.32)
Fatigue, *n* (%)	
Yes	238 (87.82)
No	33 (12.18)
Fever, *n* (%)	
Yes	229 (84.50)
No	42 (15.50)
Extramedullary infiltration, *n* (%)	
Yes	95 (35.06)
No	176 (64.94)
Hemorrhagic tendency, *n* (%)	
Yes	198 (73.06)
No	73 (26.94)
ECOG status, *n* (%)	
0	28 (10.33)
1	47 (17.34)
2	67 (24.72)
3	75 (27.68)
4	54 (19.93)
Peripheral blood blasts (%) (*M*, *Q*)	2 (0, 16.2)
Bone marrow blast (%) (*M*, *Q*)	9 (5.0, 18.8)
Myelofibrosis grade, *n* (%)	139 (51.29)
0	50 (35.97)
1	52 (37.41)
2	37 (26.62)
Lineages of marrow dysplasia, *n* (%)	
0	58 (21.40)
1	87 (32.10)
2	81 (29.89)
3	45 (16.61)
FAB classification, *n* (%)	
MD-CMML	109 (40.22)
MP-CMML	162 (59.78)
WHO classification, *n* (%)	
CMML-0	43 (15.87)
CMML-1	97 (35.79)
CMML-2	131 (48.34)

### 3.2 Laboratory findings

As shown in Table 2, the median white blood cell count (WBC) was 16.3 × 10^9^/L (range: 2.32–121.8), hemoglobin (HGB) was 72.0 g/L (range: 41–157), platelet count (PLT) was 83 × 10^9^/L (range: 11–946), and monocyte count was 3.7 × 10^9^/L (range: 0.1–49.8). Monocytes accounted for 29% (range: 6.7–73.5%) of total leukocytes, and blasts constituted 2.6% (range: 0–17%). Bone marrow analysis revealed dysplasia of bone marrow (DBM) in 223 patients (82.29%), including 97 cases (35.79%) with single-lineage DBM, 89 cases (32.84%) with two-lineage DBM, and 47 cases (17.34%) with three-lineage DBM. Bone marrow biopsy showed hypercellularity in 73 cases, hypocellularity in 59 cases, and myelofibrosis in 139 cases.

**Table 2:** Laboratory findings of 271 CMML patients.

Items and variables	Baseline distribution (*n* = 271)
WBC (×10^9^/L) (*M*, *Q*)	16.3 (6.32, 56.8)
HB (g/L) $\left(\bar{X}\pm S\right)$	72.0 ± 41
PLT (×10^9^/L) $\left(\bar{X}\pm S\right)$	83 ± 59
AMC (×10^9^/L) (*M*, *Q*)	3.7 (2.1, 11.8)
ALC (×10^9^/L) (*M*, *Q*)	3.45 (1.98, 7.35)
ANC (×10^9^/L) (*M*, *Q*)	6.2 (3.4, 24.6)
Monocytes (%) (*M*, *Q*)	29 (10.5, 43.5)
Albumin (g/L) (*M*, *Q*)	32 (28, 36)
β2-MG (mg/L) (*M*, *Q*)	4.21 (2.59, 7.83)
LDH (U/L) (*M*, *Q*)	392 (298, 786)

### 3.3 Cytogenetic and molecular genetic findings

**Figure 2:**
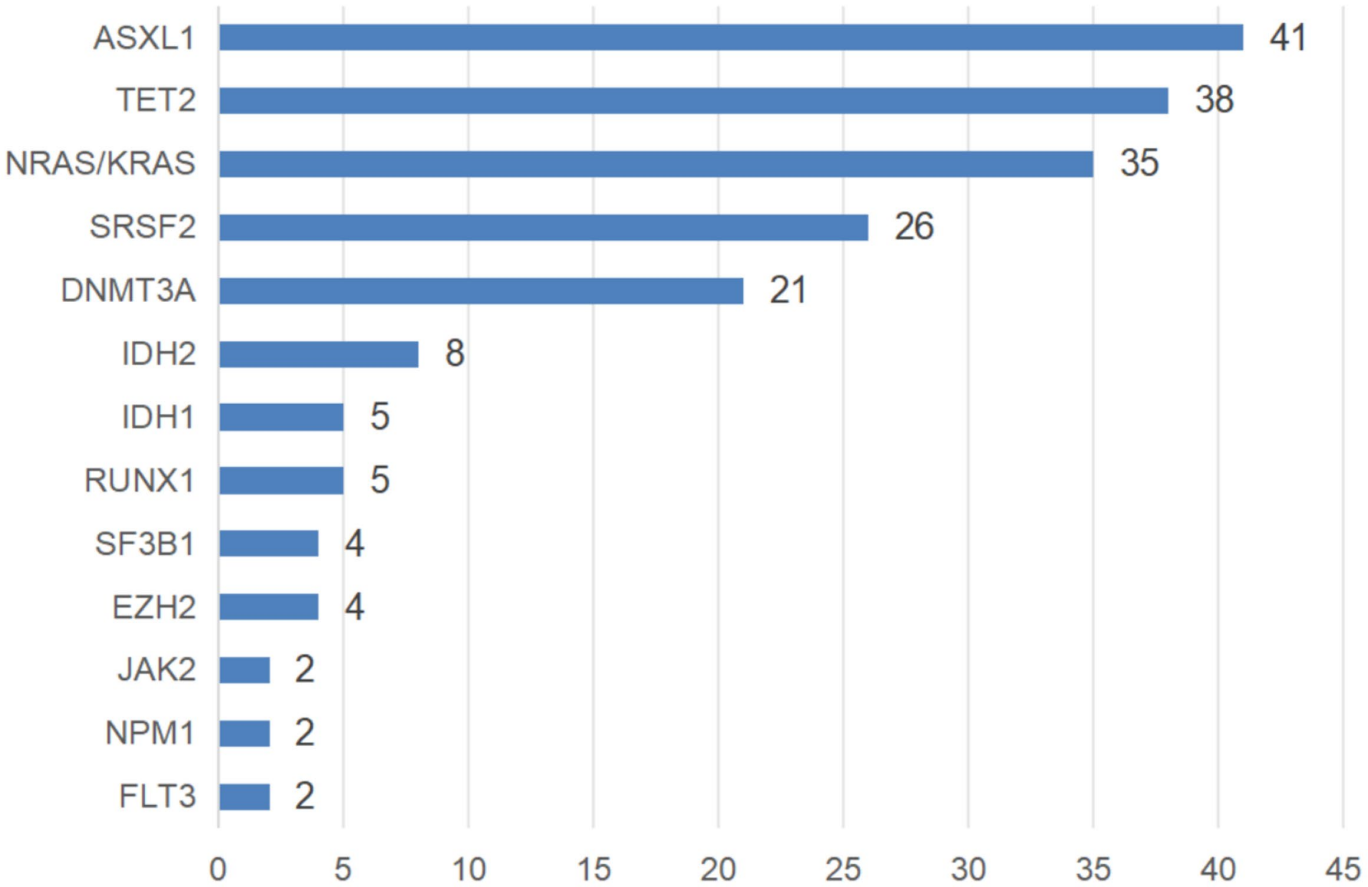
Common types of gene mutations in CMML patients.

A total of 243 patients underwent karyotyping, with chromosomal abnormalities detected in 64 cases (26.34%). These included complex karyotypes (*n* = 16), +8 (*n* = 13), del(9)(p21) (*n* = 5), del(20)(q12) (*n* = 4), 20q12- (*n* = 3), and other abnormalities (*n* = 23). Molecular genetic testing was performed in 204 patients, revealing mutations in 193 cases (94.61%). Among them, 54 patients (27.98%) had one mutation, 58 cases (30.05%) had two mutations, 51 cases (26.42%) had three mutations, 22 cases (11.40%) had four mutations, and 7 cases (3.63%) had five mutations. Commonly mutated genes are illustrated in Figure 2.

### 3.4 Morphological characteristics of CMML

**Figure 3:**
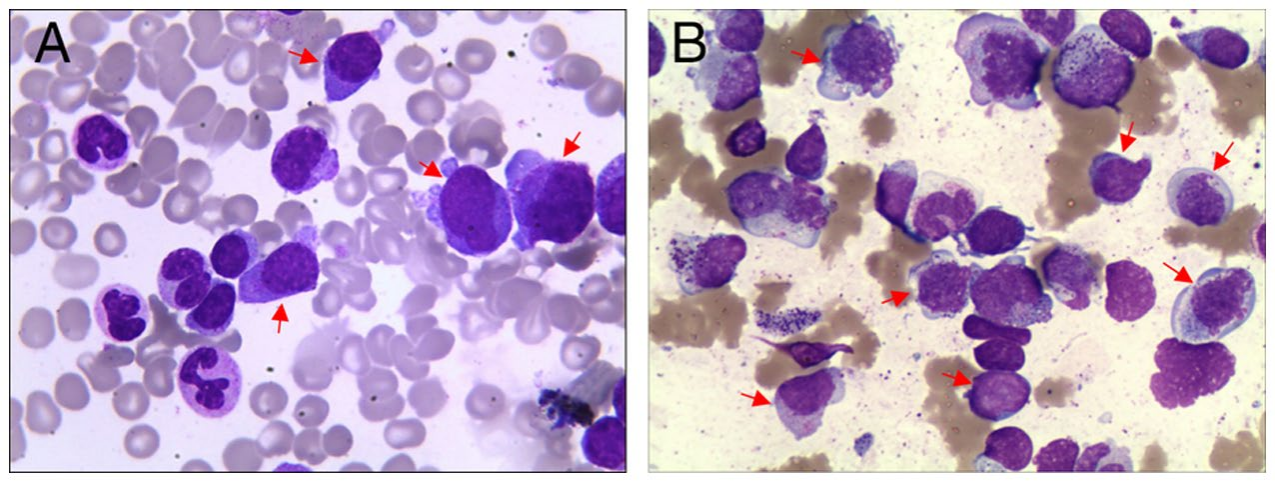
Morphological characteristics of cells in patients with CMML. **(A,B)** Wright-Giemsa staining of bone marrow (400×, immature CMML cells are now clearly indicated with red arrows).

As illustrated in Figure 3, the morphological features of bone marrow cells from CMML patients enrolled in this study are summarized as follows: (A,B) The differentiation sequences of promonocytes and monoblasts are evident, as indicated by arrows. Promonocytes exhibit large cell bodies, round or irregularly shaped nuclei, fine reticular chromatin, and clearly visible nucleoli. In comparison, immature monocytes display more pronounced nuclear folding and a slightly increased chromatin density. There is monocytic lineage hyperplasia with mild maturation disturbance; however, the proportion of blast cells (monoblasts and promonocytes) remains below 20%.

### 3.5 Histopathological characteristics of CMML

**Figure 4:**
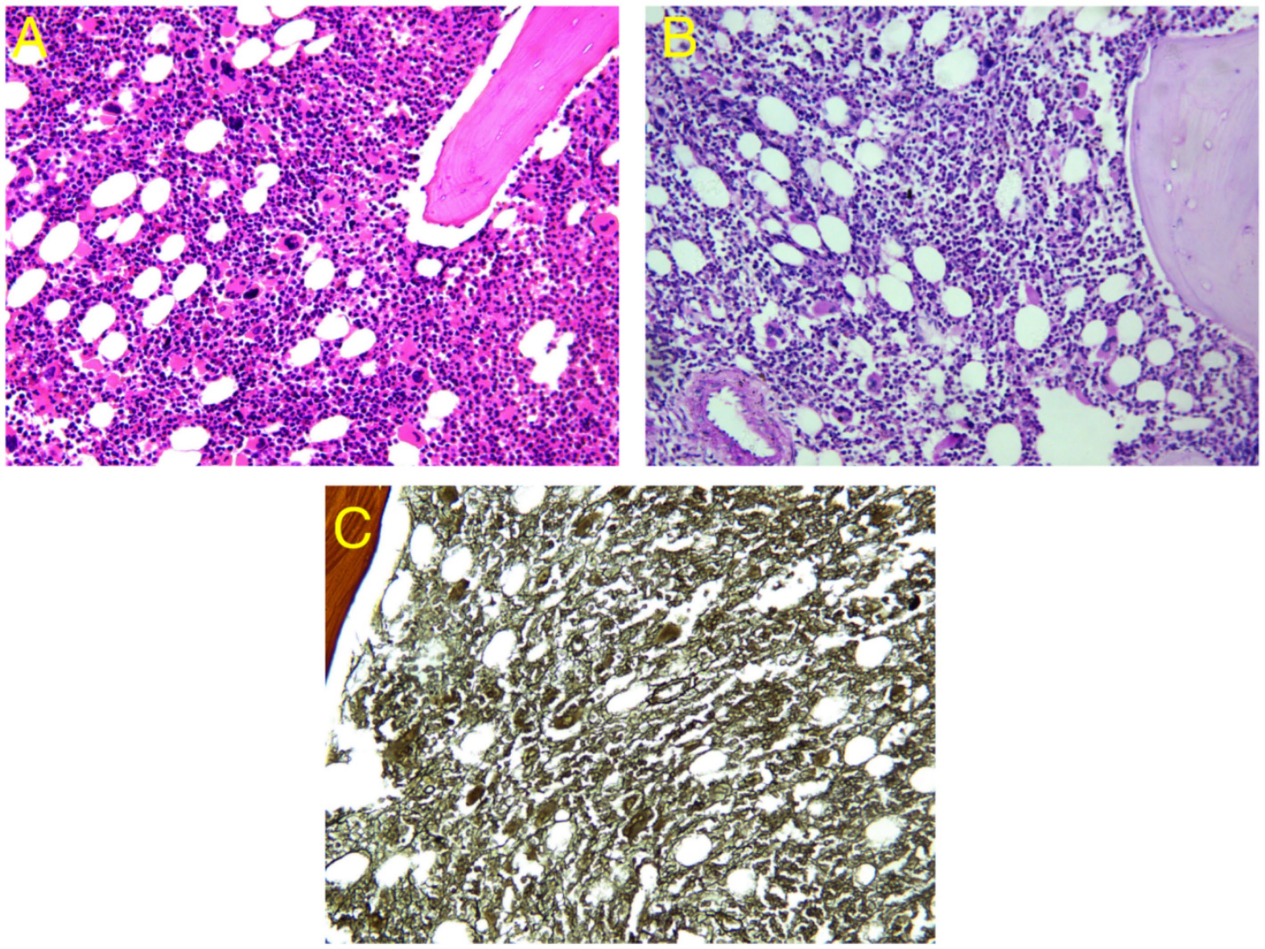
Histopathological characteristics of patients with CMML. **(A)** Bone marrow hyperplasia is markedly active, with extensive monocyte infiltration (HE staining ×20); **(B)** Immature monocytes show positive PAS staining, with visible pale red granular deposits in the cytoplasm (PAS staining ×20); **(C)** Reticular fiber staining indicates moderate fibrosis (MF-1 grade).

As shown in Figure 4, the histopathological findings of bone marrow biopsies from CMML patients included in this study are described as follows: (A) Histological examination of hematoxylin and eosin (HE)-stained sections reveals markedly hypercellular bone marrow with significant proliferation of both granulocytic and monocytic lineages. A substantial infiltration of atypical monocytes is evident. These CMML cells are typically round or oval in shape, with slightly coarse nuclear chromatin. In certain cells, distinct nucleoli are observable, and the cytoplasm is abundant. The cellular morphology is consistent with that of the monocytic lineage. (B) Periodic acid-Schiff (PAS) staining of immature monocytes is positive, with pale red granular material deposited in the cytoplasm, indicative of lysosome-like substance accumulation and supportive of monocytic differentiation. (C) Reticular fiber staining demonstrates increased reticular fibers within the bone marrow interstitium, consistent with moderate fibrosis (MF-1 grade). No dense fascicular arrangement or significant collagen deposition is observed.

### 3.6 Treatment and efficacy outcomes

**Figure 5:**
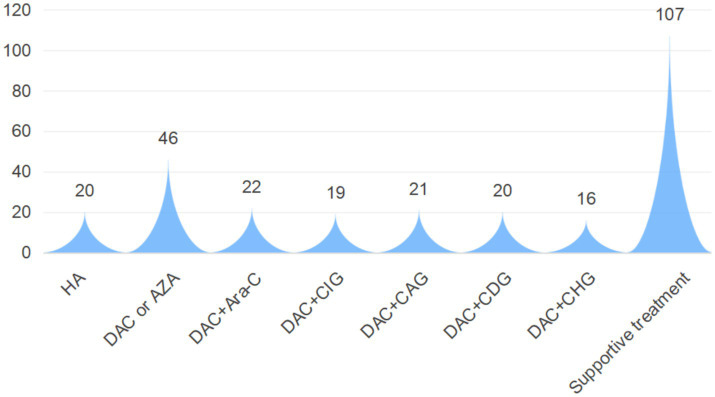
Treatment regimens for CMML patients in this study.

As shown in Figure 5, First-line therapy for CMML primarily involved chemotherapy. Except for 20 patients treated with the HA regimen [homoharringtonine + cytarabine (Ara-C)], 144 patients received regimens incorporating hypomethylating agents (HMAs). Specific regimens included DAC or AZA monotherapy, DAC + Ara-C, DAC + CIG [Ara-C + idarubicin + rhG-CSF], DAC + CAG [Ara-C + aclarubicin + rhG-CSF], DAC + CDG [Ara-C + daunorubicin + rhG-CSF], and DAC + CHG [Ara-C + homoharringtonine + rhG-CSF]. The remaining 107 patients received only symptomatic and supportive care, including leukoreduction, blood transfusion, anti-infective therapy, erythropoietin, and circulatory support. This study evaluated the therapeutic effect of CMML patients who met the treatment course norms. The results showed that a total of 199 patients could be eligible for efficacy evaluation. Among them, 97 patients achieved CR, 63 patients achieved PR, 32 patients achieved SD, and 7 patients developed PD. It is worth noting that during the follow-up period, 34 patients (12.55%) transformed into AML, among which 13 patients (38.24%) received allogeneic transplantation.

### 3.7 Survival and prognostic analysis

**Figure 6:**
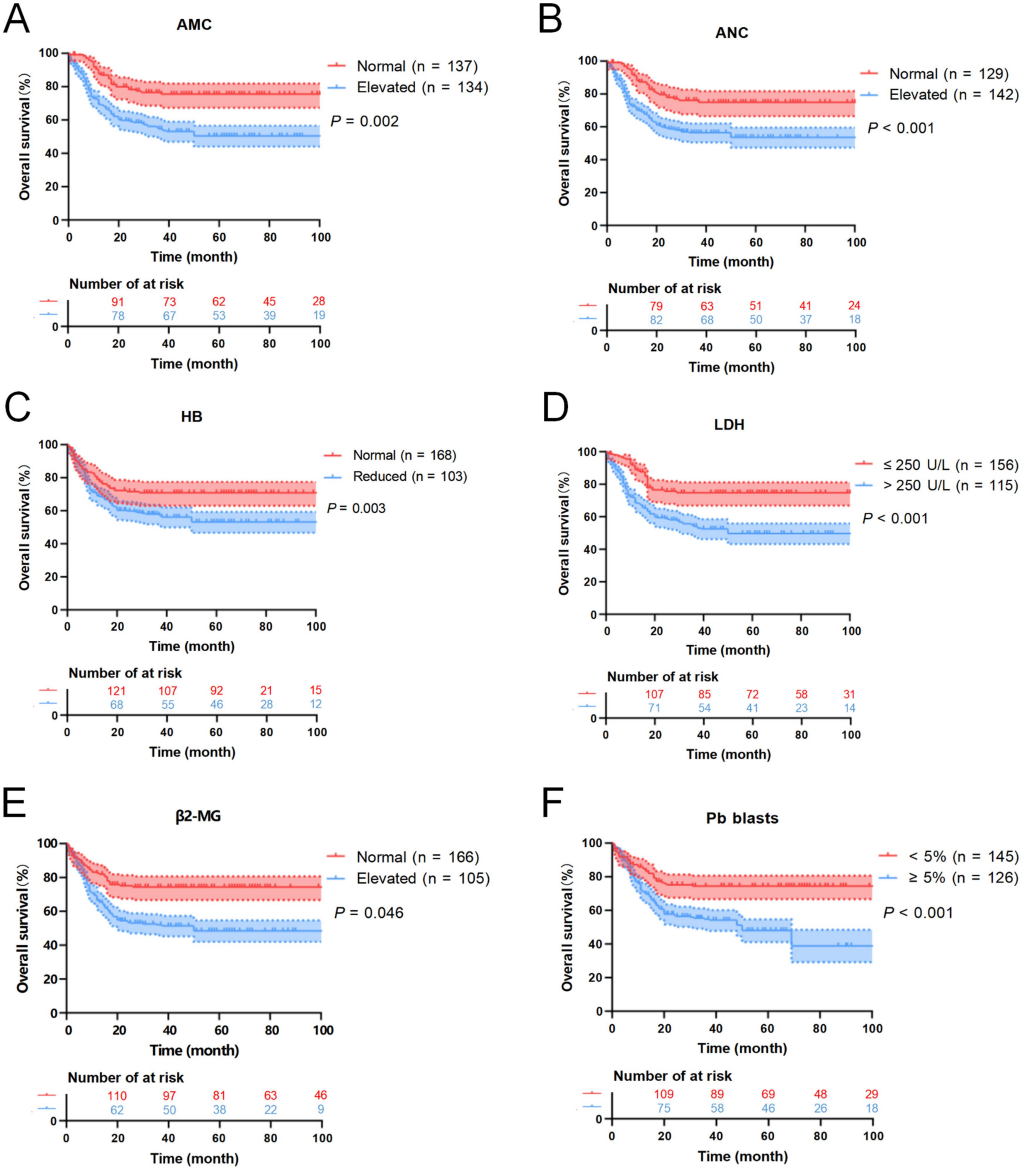
Kaplan–Meier OS curves for CMML patients. **(A)** The overall survival curves of monocyte count of CMML patients. **(B)** The overall survival curves of neutrophil count of CMML patients. **(C)** The overall survival curves of HB of CMML patients. **(D)** The overall survival curves of LDH of CMML patients. **(E)** The overall survival curves of β2-MG of CMML patients. **(F)** The overall survival curves of peripheral blood blasts of CMML patients.

Follow-up was completed by June 30, 2025. Of the 271 patients, 159 cases (58.67%) were alive, 97 cases (35.79%) had died, and 15 cases (5.54%) were lost to follow-up. The median OS was 23.5 months (range: 0.5–109). Univariate analysis was conducted to identify prognostic factors associated with OS. Log-rank test results indicated that elevated neutrophil count, increased monocyte levels, reduced HB, elevated LDH, elevated β2-MG, and peripheral blood blasts ≥5% were significantly associated with poorer OS (*p* < 0.05) (Table 3). Detailed survival curves are presented in Figure 6. These statistically significant variables were further analyzed by using the Cox proportional hazards regression model. Multivariate analysis confirmed that extramedullary involvement, reduced HB and peripheral blood blasts ≥5% were independent prognostic factors for OS in CMML patients (Table 4).

**Table 3:** Clinical characteristics of patients with CMML and univariate analysis of OS.

Evaluation indicators	1-year OS (%)	3-year OS (%)	5-year OS (%)	*χ*^2^ value	*p* value
Age (year)				6.132	0.246
≤60	69.30	53.20	41.50		
>60	61.10	51.25	39.95		
Gender				1.534	0.358
Male	70.55	62.30	48.56		
Female	71.20	64.74	53.35		
Hepatomegaly				0.289	0.523
Yes	67.05	60.25	50.40		
No	72.40	59.50	44.80		
Splenomegaly				0.195	0.587
Yes	63.60	55.40	41.10		
No	75.20	61.30	50.45		
Lymphadenopathy				0.384	0.475
Yes	68.30	59.90	42.45		
No	71.10	62.35	53.30		
Extramedullary involvement				2.217	0.071
Yes	67.20	52.05	41.56		
No	74.67	65.92	50.38		
FAB classification				0.084	0.763
MD-CMML	70.60	62.35	51.50		
MP-CMML	67.80	57.90	44.10		
WHO classification				0.578	0.452
CMML-0	74.20	68.25	60.40		
CMML-1	70.15	61.40	53.50		
CMML-2	64.20	54.10	41.65		
WBC				0.251	0.523
Normal	68.60	59.35	47.50		
Elevated	64.83	54.15	40.25		
AMC				10.396	**0.002**
Normal	69.27	57.47	45.25		
Elevated	63.56	51.05	39.55		
ALC				1.779	0.183
Normal	70.05	63.90	49.76		
Elevated	62.47	55.28	38.63		
ANC				9.533	**<0.001**
Normal	67.27	55.47	39.53		
Elevated	63.56	51.05	35.21		
HB				10.872	**0.003**
Normal	76.05	68.90	54.76		
Reduced	69.50	59.20	44.66		
PLT				1.313	0.252
Normal	76.45	66.30	58.46		
Reduced	72.20	61.00	49.36		
LDH				8.163	**<0.001**
≤250 U/L	72.50	59.46	46.15		
>250 U/L	65.30	52.59	35.65		
ECOG status				0.984	0.365
<2	75.22	63.48	54.46		
≥2	70.20	59.00	45.38		
Albumin				0.144	0.725
Normal	73.60	61.35	51.08		
Reduced	61.05	52.38	38.56		
β2-MG				5.367	**0.046**
Normal	74.58	65.05	48.80		
Elevated	64.22	54.76	36.19		
Peripheral blood blasts				12.584	**<0.001**
<5%	73.45	62.24	47.59		
≥5%	64.30	48.90	36.51		
Bone marrow blasts				0.281	0.465
<10%	71.50	61.35	48.34		
≥10%	67.25	52.83	41.56		
Treatment regimen				3.354	0.085
HMA	75.40	64.15	53.78		
Non-HMA	63.05	54.88	42.90		

The bold values indicate that the *p* value is < 0.05, meaning the difference is statistically significant.

**Table 4:** Multivariate analysis of OS in CMML patients with COX proportional hazards.

Index	*p* value	*HR*	95%CI	
			Lower limit	Upper limit
Age	0.325	1.217	0.697	1.685
Gender	0.414	1.514	0.650	2.784
Hepatomegaly	0.658	1.462	0.673	2.217
Splenomegaly	0.116	1.023	0.523	1.579
Lymphadenopathy	0.589	1.235	0.617	2.563
Extramedullary involvement	**0.035**	1.824	1.373	2.629
FAB classification	0.802	1.326	0.585	2.557
WHO classification	0.260	0.780	0.256	1.586
WBC	0.865	0.726	0.364	1.283
AMC	0.169	1.227	0.812	1.896
ALC	0.582	1.143	0.756	1.689
ANC	0.524	1.158	0.720	1.678
HB	**0.043**	0.745	0.522	0.948
PLT	0.288	1.584	0.698	2.585
LDH	0.123	1.658	0.795	3.468
ECOG	0.455	1.132	0.685	1.711
Albumin	0.612	1.160	0.729	1.918
β2-MG	0.184	1.131	0.586	1.993
Peripheral blood blasts	**0.023**	2.526	1.682	3.528
Bone marrow blasts	0.834	1.157	0.693	2.573
Treatment regimen	0.182	1.874	0.737	2.477

The bold values indicate that the *p* value is < 0.05, meaning the difference is statistically significant.

### 3.8 Survival analysis and comparison based on different treatment methods

**Figure 7:**
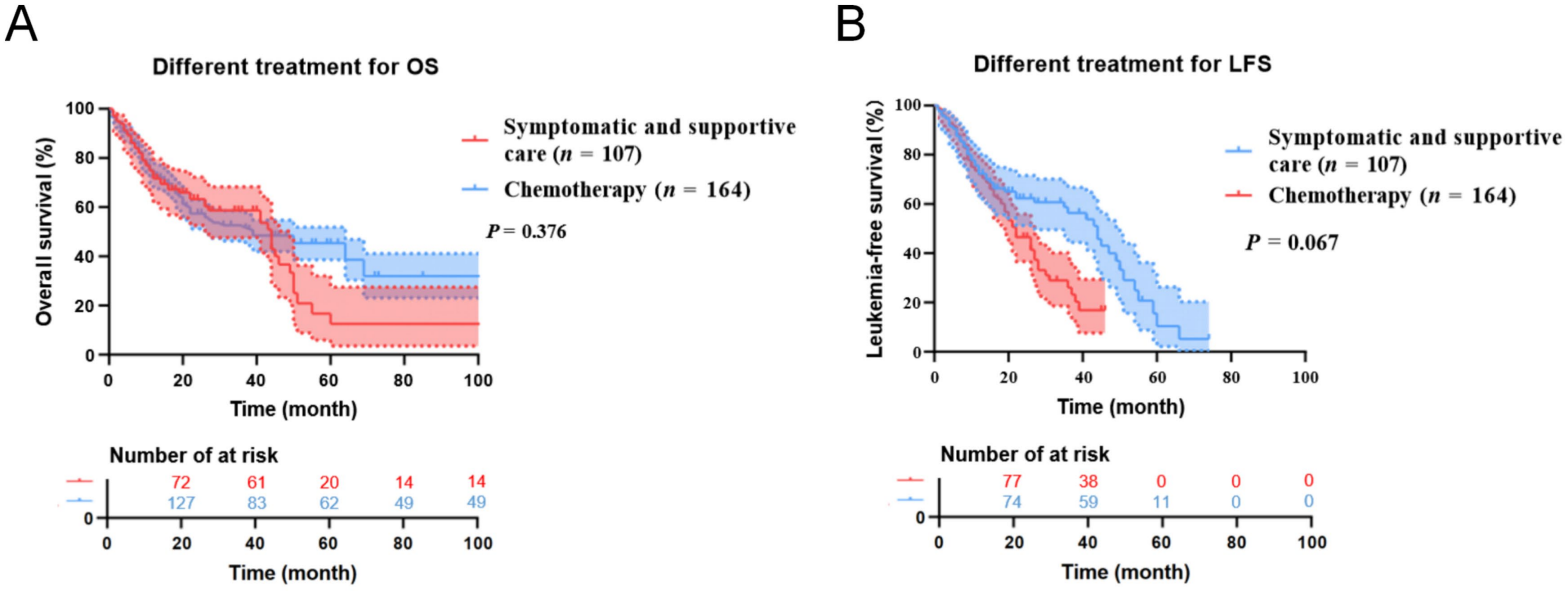
Kaplan–Meier OS and LFS curves for different treatments in CMML patients. **(A)** The overall survival curves of treatment of CMML patients. **(B)** The leukemia free survival curves of treatment of CMML patients.

Due to the differences in treatment methods among the CMML patients included in this study, the patients were divided into two groups: those receiving different chemotherapy regimens (164 cases) and those receiving only symptomatic and supportive treatment (107 cases). OS and LFS were analyzed and compared for them (Figure 7), and prognostic factor analysis was conducted for each group of patients, respectively. Multivariate analysis confirmed that extramedullary involvement, reduced WBC, reduced HB and peripheral blood blasts ≥5% were independent prognostic factors for OS in CMML patients who received chemotherapy (Table 5), while extramedullary involvement, reduced HB, peripheral blood blasts ≥5%, and bone marrow blasts ≥10% were independent prognostic factors for LFS in CMML patients who received chemotherapy (Table 6). Correspondingly, extramedullary involvement, reduced WBC and peripheral blood blasts ≥5% were independent prognostic factors for OS in CMML patients who received symptomatic and supportive care (Table 7), and elevated age, extramedullary involvement, reduced HB, elevated ECOG and peripheral blood blasts ≥5% were independent prognostic factors for LFS in CMML patients who received symptomatic and supportive care (Table 8).

**Table 5:** Multivariate analysis of OS in CMML patients who received chemotherapy with COX proportional hazards.

Index	*p* value	*HR*	95%CI	
			Lower limit	Upper limit
Age	0.335	1.123	0.497	1.977
Gender	0.534	1.646	0.834	2.353
Hepatomegaly	0.642	1.513	0.675	2.164
Splenomegaly	0.196	1.183	0.563	1.524
Lymphadenopathy	0.722	1.324	0.688	2.478
Extramedullary involvement	**0.028**	1.736	1.321	2.345
FAB classification	0.874	1.343	0.596	2.542
WHO classification	0.262	0.797	0.278	1.657
WBC	**0.043**	0.677	0.356	0.935
AMC	0.754	1.322	0.856	1.812
ALC	0.432	1.123	0.744	1.654
ANC	0.654	1.138	0.756	1.743
HB	**0.027**	0.564	0.227	0.824
PLT	0.533	1.244	0.699	1.956
LDH	0.586	1.643	0.726	2.969
ECOG	0.257	1.531	0.685	2.143
Albumin	0.965	0.745	0.354	1.145
β2-MG	0.245	1.145	0.757	1.875
Peripheral blood blasts	**0.039**	2.373	1.732	3.148
Bone marrow blasts	0.865	1.154	0.793	1.735
Treatment regimen	0.136	1.757	0.737	2.598

The bold values indicate that the *p* value is < 0.05, meaning the difference is statistically significant.

**Table 6:** Multivariate analysis of LFS in CMML patients who received chemotherapy with COX proportional hazards.

Index	*p* value	*HR*	95%CI	
			Lower limit	Upper limit
Age	0.415	1.054	0.388	1.985
Gender	0.642	1.532	0.755	2.643
Hepatomegaly	0.294	1.323	0.753	2.312
Splenomegaly	0.846	1.021	0.624	1.439
Lymphadenopathy	0.574	1.511	0.822	2.231
Extramedullary involvement	**0.007**	1.526	1.158	2.126
FAB classification	0.635	1.343	0.645	2.232
WHO classification	0.524	0.832	0.243	1.477
WBC	0.426	0.845	0.565	1.186
AMC	0.524	1.342	0.645	1.947
ALC	0.735	1.147	0.856	1.589
ANC	0.573	1.328	0.675	2.213
HB	**0.025**	0.679	0.236	0.956
PLT	0.853	1.549	0.798	2.476
LDH	0.733	1.343	0.866	1.915
ECOG	0.521	1.643	0.876	2.367
Albumin	0.723	0.685	0.165	1.036
β2-MG	0.143	1.101	0.797	1.793
Peripheral blood blasts	**0.037**	2.123	1.768	2.604
Bone marrow blasts	**0.049**	1.688	0.955	2.462
Treatment regimen	0.423	1.843	0.854	2.775

The bold values indicate that the *p* value is < 0.05, meaning the difference is statistically significant.

**Table 7:** Multivariate analysis of OS in CMML patients who received symptomatic and supportive care with COX proportional hazards.

Index	*p* value	*HR*	95%CI	
			Lower limit	Upper limit
Age	0.125	1.184	0.695	1.733
Gender	0.214	1.297	0.742	1.756
Hepatomegaly	0.586	1.674	0.839	2.264
Splenomegaly	0.224	1.233	0.689	1.879
Lymphadenopathy	0.742	1.632	0.817	2.486
Extramedullary involvement	**0.014**	1.839	1.268	2.526
FAB classification	0.462	1.263	0.785	1.857
WHO classification	0.635	0.836	0.376	1.274
WBC	**0.045**	0.626	0.263	0.975
AMC	0.268	1.324	0.543	2.195
ALC	0.578	1.244	0.788	1.767
ANC	0.596	1.642	0.886	2.487
HB	0.068	0.567	0.255	0.876
PLT	0.085	1.049	0.798	1.485
LDH	0.213	1.158	0.695	1.762
ECOG	0.425	1.232	0.694	1.951
Albumin	0.656	0.760	0.328	1.113
β2-MG	0.234	1.256	0.686	1.953
Peripheral blood blasts	**0.023**	2.526	1.682	3.528
Bone marrow blasts	0.674	1.177	0.745	1.678
Treatment regimen	0.283	1.575	0.787	2.453

The bold values indicate that the *p* value is < 0.05, meaning the difference is statistically significant.

**Table 8:** Multivariate analysis of LFS in CMML patients who received symptomatic and supportive care with COX proportional hazards.

Index	*p* value	*HR*	95%CI	
			Lower limit	Upper limit
Age	**0.028**	1.513	1.174	2.134
Gender	0.514	1.465	0.758	2.086
Hepatomegaly	0.554	1.362	0.724	1.916
Splenomegaly	0.216	1.235	0.624	1.678
Lymphadenopathy	0.682	1.637	0.817	2.364
Extramedullary involvement	**0.027**	1.648	1.122	2.245
FAB classification	0.756	1.425	0.785	2.358
WHO classification	0.436	0.870	0.467	1.456
WBC	0.854	0.657	0.364	1.183
AMC	0.258	1.126	0.712	1.753
ALC	0.591	1.243	0.856	1.767
ANC	0.584	1.158	0.784	1.778
HB	**0.043**	0.576	0.225	0.854
PLT	0.173	1.282	0.718	1.685
LDH	0.324	1.458	0.736	2.260
ECOG	**0.045**	2.127	1.586	2.885
Albumin	0.514	0.610	0.397	1.127
β2-MG	0.142	1.142	0.685	1.784
Peripheral blood blasts	**0.023**	2.526	1.682	3.528
Bone marrow blasts	0.234	1.156	0.743	1.585
Treatment regimen	0.366	1.475	0.787	2.268

The bold values indicate that the *p* value is < 0.05, meaning the difference is statistically significant.

### 3.9 Analysis of risk factors for mortality

**Figure 8:**
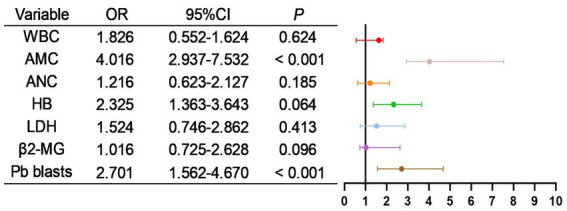
Multivariate logistic regression analysis of risk factors for mortality in CMML patients.

As shown in Table 9, all potentially relevant prognostic factors were included in univariate logistic regression analysis. Results indicated that WBC, absolute monocyte count (AMC), absolute neutrophil count (ANC), HB, LDH, β2-MG, and peripheral blood blasts were significantly associated with patient mortality (all *p* < 0.05). Besides, as shown in Figure 8, factors with *p* < 0.05 in the univariate analysis were included in multivariate analysis, revealing that AMC, Pb blasts were independent risk factors for mortality.

**Table 9:** Univariate analysis of risk factors for death in CMML patients.

Causes of death and related factors	*B*	S.E.	Wald	df	*p*	Exp (*B*)	95.0% CI for Exp (*B*)	
							Lower	Upper
Age	−0.048	0.278	0.074	1	0.743	0.858	0.466	1.354
Gender	0.644	0.243	3.754	1	0.068	1.643	0.854	2.356
Hepatomegaly	−0.074	0.532	0.067	1	0.769	0.854	0.323	1.455
Splenomegaly	0.123	0.433	0.325	1	0.722	1.354	0.855	1.965
Lymphadenopathy	0.067	0.329	0.223	1	0.636	1.213	0.766	1.876
Extramedullary involvement	0.758	0.453	4.247	1	0.062	2.232	1.445	3.326
FAB classification	0.323	0.431	1.543	1	0.167	1.542	0.754	2.885
WHO classification	0.256	0.341	1.233	1	0.143	1.631	0.765	2.354
WBC	1.643	0.532	21.243	1	**0.030**	3.876	2.132	5.242
AMC	0.412	0.321	4.522	1	**0.038**	1.521	1.311	2.188
ALC	0.635	0.764	8.332	1	0.086	2.225	0.975	4.123
ANC	0.513	0.531	9.133	1	**0.014**	1.643	1.132	2.754
HB	1.872	0.423	27.453	1	**<0.001**	6.163	3.633	11.463
PLT	−0.075	0.341	0.012	1	0.964	0.879	0.584	1.653
LDH	0.654	0.542	5.467	1	**0.027**	1.798	1.134	2.785
ECOG	0.543	0.714	2.378	1	0.216	1.776	0.985	1.865
Albumin	0.112	0.376	1.047	1	0.108	1.533	0.957	2.533
β2-MG	0.523	0.251	6.750	1	**0.006**	1.734	1.193	2.654
Peripheral blood blasts	1.089	0.234	21.348	1	**<0.001**	2.875	1.837	4.780
Bone marrow blasts	0.647	0.846	6.482	1	0.073	2.534	1.233	4.023
Treatment regimen	−0.083	0.456	0.521	1	0.852	1.032	0.634	1.857

The bold values indicate that the *p* value is < 0.05, meaning the difference is statistically significant.

## 4 Discussion

CMML is a relatively rare non-solid malignant clonal disorder of the hematopoietic system encountered in clinical practice. It is characterized by abnormal bone marrow hematopoiesis, persistent and marked monocytosis in peripheral blood—typically exceeding threefold the normal level—and frequently accompanied by anemia and thrombocytopenia. The disease also exhibits features of MDS, such as dysplasia, as well as characteristics of MPN, including clonal expansion (8, 9). Epidemiological studies have indicated that CMML predominantly affects middle-aged and elderly males. Clinical manifestations are generally nonspecific and highly heterogeneous, ranging from fatigue, abnormal blood counts, anemia, infections, bleeding, weight loss, night sweats, to hepatosplenomegaly or lymphadenopathy (10–12). In this study, a total of 271 CMML patients were enrolled, with a male-to-female ratio of 1.91:1 and a median age at diagnosis of 66 years. The primary clinical symptoms or signs observed at initial diagnosis included abnormal blood counts, fever, infection, fatigue, abdominal discomfort, bleeding, and hepatosplenomegaly, which align with the current international epidemiological profile of CMML.

Clinically, CMML is associated with a generally poor prognosis, with approximately one-quarter of patients progressing to acute myeloid leukemia (AML) or succumbing to disease progression within a few years (13). Due to the high heterogeneity of CMML in terms of clinical presentation, morphology, treatment response, and prognosis, there remains a lack of consensus or standardized criteria for disease assessment and prognostic evaluation. Therapeutic options for CMML remain limited and primarily include allogeneic hematopoietic stem cell transplantation, epigenetic agents such as HMAs, and supportive care measures such as leukocytoreduction and blood component transfusion. However, these interventions do not offer curative potential (14).

As previously mentioned, in addition to the clinical heterogeneity that complicates the diagnosis and management of CMML, laboratory findings also pose diagnostic challenges. In this study, 223 patients (82.29%) exhibited bone marrow dysplasia, and 139 patients were diagnosed with myelofibrosis. These findings underscore the difficulty in distinguishing CMML from MDS or MPN based solely on morphological and histopathological bone marrow evaluations. Therefore, comprehensive diagnostic approaches incorporating morphological, immunophenotypic, cytogenetic, and molecular (MICM) analyses are essential. Previous studies (15) have demonstrated that nearly one-third of CMML patients exhibit cytogenetic abnormalities. Chromosomal aberrations not only aid in differential diagnosis but also serve as important prognostic indicators, particularly regarding the risk of AML transformation and overall survival. Research (16) has identified complex karyotypes as the most frequently observed chromosomal abnormalities in CMML, followed by +21, −7/del(7q), del(20q), i(17q), and −17/del(17p). Acquired cytogenetic abnormalities (ACAs) are more commonly detected in patients initially presenting with normal or low-risk karyotypes and are significantly associated with AML progression and reduced survival duration. Additional studies (17) have shown that patients with abnormal karyotypes generally exhibit shorter overall survival, poorer prognosis, and higher risk of leukemic transformation. Furthermore, a stratified risk assessment model based on cytogenetic abnormalities (18) revealed that patients in the low-risk group had significantly better median OS compared to those in the intermediate- and high-risk groups, with a marked difference observed in the high-risk cohort. Notably, patients with more than three chromosomal abnormalities exhibited significantly shorter OS, highlighting the importance of cytogenetic data in CMML risk stratification.

Molecular abnormalities are detectable in the majority of CMML patients. Although these abnormalities are not exclusive to CMML, they play a significant role in disease progression and prognosis. For instance, NRAS mutations have been associated with reduced overall survival and disease-free survival, elevated white blood cell counts, decreased platelet levels, and the presence of blasts in peripheral blood, indicating a poor prognosis (19). A study (20) established a “BLAST” survival model for CMML and identified PHF6 and TET2 mutations as favorable prognostic factors. Conversely, mutations in DNMT3A, U2AF1, BCOR, SETBP1, ASXL1, NRAS, PTPN11, RUNX1, and TP53 were identified as adverse prognostic indicators. Moreover, research (21) has indicated that approximately 14% of CMML patients may exhibit an indolent disease course, which is associated with a relatively favorable prognosis. These patients typically present with higher hemoglobin and platelet levels, lower JAK2 mutation frequency, and less pronounced cytopenias. In contrast, urgent therapeutic intervention is warranted in patients presenting with leukocytosis, elevated lymphocyte/monocyte counts, increased circulating immature cells or blasts, elevated bone marrow blast percentages, and specific gene mutations such as NRAS, ASXL1, and RUNX1. Early identification of these risk factors is crucial for timely therapeutic adjustments and improved patient outcomes.

Despite limited therapeutic advancements in CMML, certain interventions have shown promise. While epigenetic therapies and allo-HSCT have modestly improved patient outcomes, the overall therapeutic efficacy and long-term survival rates remain suboptimal. A study (22) demonstrated that the combination of HMAs and venetoclax can enhance treatment response and prognosis in CMML patients. However, caution is warranted with the use of granulocyte colony-stimulating factor (G-CSF), as it may lead to severe complications such as abdominal pain and splenic infarction. Therefore, clinicians should remain vigilant regarding rare adverse effects and consider individualized treatment strategies (23). Recent investigations into JAK/STAT inhibitors have shown encouraging results. Ruxolitinib, a JAK1/2 inhibitor, has demonstrated acceptable safety and potential efficacy in CMML patients, while other agents such as momelotinib and pacritinib are currently under clinical evaluation (24, 25). Additionally, targeted anti-GM-CSF monoclonal antibody therapy (lenzilumab) has shown favorable tolerability and minimal side effects, with ongoing studies exploring its combination with HMAs (26). The novel oral PLK1 inhibitor onvansertib has also shown therapeutic potential for patients with relapsed, refractory, or comorbid CMML (27).

Notably, IMM01 (Timdarpacept), a SIRPα-Fc fusion protein targeting CD47, has demonstrated promising anti-tumor activity. By blocking the CD47/SIRPα “do not eat me” signal, IMM01 enables macrophages to recognize and phagocytose tumor cells while simultaneously activating the Fcγ receptor to transmit an “eat me” signal. Given that CMML pathogenesis involves both abnormal hematopoietic stem cell proliferation and immune evasion, azacitidine primarily targets tumor cell DNA methylation, altering their epigenetic state and enhancing immune recognition. IMM01 complements this mechanism by modulating the immune system to enhance tumor cell clearance. The combination of azacitidine and IMM01 may synergistically target CMML cells through multiple mechanisms, potentially leading to improved therapeutic outcomes (28).

## 5 Conclusion

In summary, due to the rarity and clinical heterogeneity of CMML, its manifestations and disease characteristics often lack specificity, resulting in a lack of consensus on diagnostic, therapeutic, and prognostic approaches. Future research should focus on multi-center collaborative studies involving larger patient cohorts to develop comprehensive and effective diagnostic, treatment, and prognostic models. Concurrently, further investigations into the epigenetic, molecular, and cytogenetic mechanisms underlying CMML are warranted to identify novel therapeutic targets. The development and clinical evaluation of new therapeutic agents are essential to improve patient outcomes.

## Data Availability

The original contributions presented in the study are included in the article/supplementary material, further inquiries can be directed to the corresponding authors.
